# Transvaginal in-bag directional morcellation with endometrial preservation for large uteri: an initial clinical feasibility study

**DOI:** 10.3389/fmed.2026.1910837

**Published:** 2026-09-09

**Authors:** Chang Zhou, Huan Yu, Shiyu Zhang, Jing Liang, Bin Ling

**Affiliations:** 1 Chinese Academy of Medical Sciences & Peking Union Medical College, Beijing, China; 2 Department of Obstetrics and Gynecology, China-Japan Friendship Hospital, Beijing, China

**Keywords:** directional morcellation, endometrial integrity, in-bag morcellation, laparoscopic total hysterectomy, large uterus

## Abstract

**Background:**

To explore the initial clinical feasibility and preliminary surgical safety of transvaginal four-wall directional in-bag morcellation for extracting exceedingly large uteri during laparoscopic total hysterectomy (TLH), aiming to preserve endometrial integrity as an anatomical barrier.

**Methods:**

This retrospective study included 25 patients with large uteri (12-18 gestational weeks) who underwent TLH. Following uterine detachment, a transvaginal isolation bag was inserted to establish a pneumo-bag. Under direct visualization, an electromechanical morcellator performed directional volume reduction on the four myometrial walls. The endometrial layer was strictly preserved. The intact central “core cavity” and morcellated fragments were then extracted en bloc within the bag.

**Results:**

All 25 surgeries succeeded without conversion to laparotomy. Mean uterine volume was 725.6 ± 215.4 cm^3^; mean in-bag morcellation time was 12.5 ± 4.2 min. Postoperative specimen examinations confirmed macroscopic physical integrity of the endometrial cavity in this preliminary cohort. Methylene blue pressure tests showed 100% bag water-tightness. Macroscopically, zero cases of tissue spillage, vaginal wall injury, or postoperative infection occurred.

**Conclusions:**

Transvaginal directional in-bag morcellation appears to be a technically feasible and initially safe surgical option. By establishing a “dual barrier”—an intact endometrial cavity (anatomical) and a sealed bag (physical)—this novel technique shows potential in providing macroscopic physical containment. However, lacking cytological evaluation, long-term follow-up, and a comparative control group, its potential oncological safety benefits remain strictly theoretical at this preliminary stage.

## 1 Introduction

With the popularization of Enhanced Recovery After Surgery (ERAS) protocols and advancements in minimally invasive instruments, Laparoscopic Total Hysterectomy (TLH) combined with Natural Orifice Specimen Extraction (NOSE) has become a major trend in minimally invasive gynecology, offering significant advantages such as the avoidance of abdominal incisions, reduced postoperative pain, and improved cosmetic outcomes (1). However, in clinical practice, when dealing with an exceedingly large uterus (equivalent to >12 or even >16 weeks of gestation) caused by multiple uterine fibroids or severe adenomyosis, the contradiction between the limited pelvic operative space and the massive specimen becomes prominent. Consequently, transvaginal specimen extraction often becomes one of the most time-consuming and challenging steps of the procedure.

Traditional specimen extraction strategies primarily rely on colpotomy, excessive traction, or blind mechanical volume reduction (e.g., manual fragmentation, wedge resection). These methods may not only increase surgical difficulty and prolong operative time but also carry the risk of adjacent organ injury (such as the vaginal wall, rectum, or bladder) due to blind maneuvers in confined spaces. Furthermore, traditional volume reduction techniques harbor a potential safety hazard—the iatrogenic dissemination of uterine cavity contents and tissue fragments (2–4). During open morcellation or forceful physical compression, the intrauterine pressure surges, creating a risk for intrauterine fluid containing active endometrial cells or lesional tissue to reflux or spill into the abdominopelvic cavity and vaginal wound. Such disordered tissue spillage may trigger pelvic endometriosis or disseminated peritoneal leiomyomatosis (DPL), subsequently affecting the patient's postoperative quality of life. Most studies indicate that adenomyosis and multiple uterine fibroids increase the risk of developing endometrial cancer (5). If a patient harbors an undiagnosed occult endometrial cancer or uterine sarcoma, this spillage could lead to the implantation and metastasis of malignant cells in the abdominal cavity and vaginal wall, resulting in unexpected disease upstaging. This contradicts the fundamental “oncological safety” principles of gynecologic oncology and has aroused widespread concern and discussion within the international gynecologic community (4, 6–10). Particularly concerning uncontained laparoscopic power morcellation, the United States Food and Drug Administration (FDA) has issued strict safety communications and a black-box warning. The FDA strongly discourages the use of unprotected electromechanical morcellators during hysterectomy or myomectomy due to the significant risk of spreading unsuspected occult malignancies, such as uterine sarcomas, throughout the abdominopelvic cavity. This pivotal warning has profoundly reshaped the surgical perspectives on laparoscopic uterine morcellation. The current consensus within the gynecologic community dictates that to adhere to the principles of “oncological safety”, uncontained morcellation must be strictly avoided. If tissue volume reduction is required, it must be performed within a sealed containment system (in-bag morcellation). However, traditional transabdominal in-bag morcellation still faces inherent challenges, such as restricted operative space, poor ergonomics, and the potential risk of bag rupture during mechanical cutting. Consequently, the preservation of the intact endometrial cavity should not merely be considered a technical refinement for specimen extraction, but a proactive physical containment strategy aimed at establishing an additional “anatomical barrier” to minimize potential tissue spillage. Furthermore, from a histopathological perspective, maintaining the structural integrity of the endometrium is of paramount importance. An intact “core cavity” allows pathologists to comprehensively and accurately recognize, map, and sample the endometrial tissue, thereby minimizing the risk of missing focal or occult malignancies.

How to smoothly and effectively extract an exceedingly large uterine specimen while adhering to the premise of minimizing spillage remains a current clinical challenge in minimally invasive gynecology. Based on this, to address the limitations of traditional unprotected volume reduction, our research team proposed an exploratory surgical modification: “Transvaginal four-wall directional morcellation within a sealed specimen bag.” This proposed technique attempts to incorporate two core strategies: first, restricting the entire morcellation process within a sealed bag to construct a physical barrier, aiming to reduce direct contact between tissue fragments and the abdominopelvic cavity; second, shifting from the disordered nature of traditional uterine fragmentation to a directional morcellation approach characterized by “peripheral volume reduction while preserving the uterine cavity.” By resecting the excess tissue around the uterine myometrium while maximally maintaining the structural integrity of the endometrium and uterine cavity, this approach aims to establish an anatomic barrier to mitigate the risk of endogenous intrauterine fluid spillage.

This study aimed to retrospectively analyze the clinical data of the first cohort of patients at our center who underwent this technique for exceedingly large uteri (>12 weeks of gestation size) to retrospectively evaluate the initial clinical feasibility and preliminary safety profile in a pilot cohort. Through this study, we expect to explore the clinical value of this technique in constructing a protective extraction environment, hoping to provide a feasible reference scheme for the minimally invasive extraction of massive uterine specimens with strict physical containment.

## 2 Materials and methods

### 2.1 General data

This retrospective study included 25 patients with exceedingly large uteri who underwent TLH at the China-Japan Friendship Hospital between January 2025 and January 2026. Inclusion criteria were: (1) clear indications for laparoscopic total hysterectomy; (2) preoperative ultrasound or pelvic MRI estimating a uterine volume equivalent to 12–18 weeks of gestation; and (3) patient consent for the application of the in-bag morcellation technique. Exclusion criteria were: (1) preoperatively diagnosed or highly suspected uterine malignancies via imaging or pathology; (2) severe closure of the vaginal fornix or severe pelvic stenosis; and (3) specific extreme anatomical constraints precluding the safe preservation of the myometrial mantle, such as extensive submucosal myomas where preoperative MRI demonstrates an unidentifiable/indistinguishable endometrial cavity line.

To ensure surgical consistency while reflecting real-world reproducibility, all 25 procedures were performed by multiple qualified attending-level physicians within the same highly experienced surgical team.

The mean age of the cohort was 46.5 ± 5.2 years. The preoperative uterine volume was calculated based on three-dimensional measurements obtained from pelvic ultrasound or MRI, using the standard prolate ellipsoid formula: Volume (cm^3^) = length (cm) × width (cm) × anteroposterior diameter (cm) × 0.523 (11). The mean preoperative estimated uterine volume was 725.6 ± 215.4 cm^3^ (range: 400 cm^3^–1250 cm^3^).

### 2.2 Surgical technique and core steps (dual-barrier strategy)

**Figure 1:**
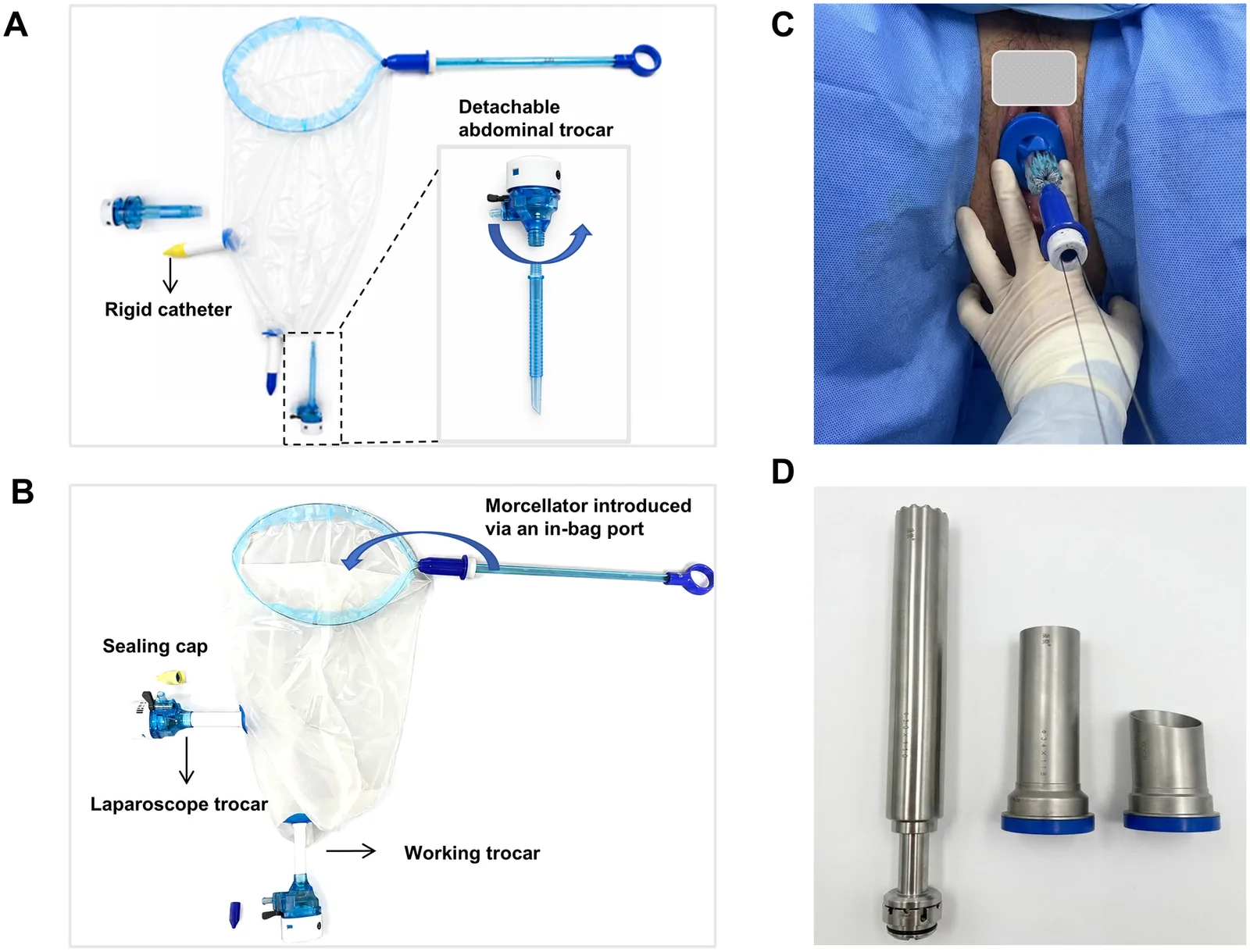
The high-strength isolation bag system and associated instruments used in the study. **(A)** The uninflated specimen containment bag featuring a rigid catheter and a detachable abdominal trocar. **(B)** The fully assembled in-bag insufflation system, highlighting the sealing cap, laparoscope channel, and working channel port. **(C)** Clinical photograph demonstrating the transvaginal establishment of the sealed containment bag (with the bag opening pulled out and fixed at the vulva). **(D)** Components of the electromechanical morcellator utilized for directional volume reduction within the bag.

**Figure 2:**
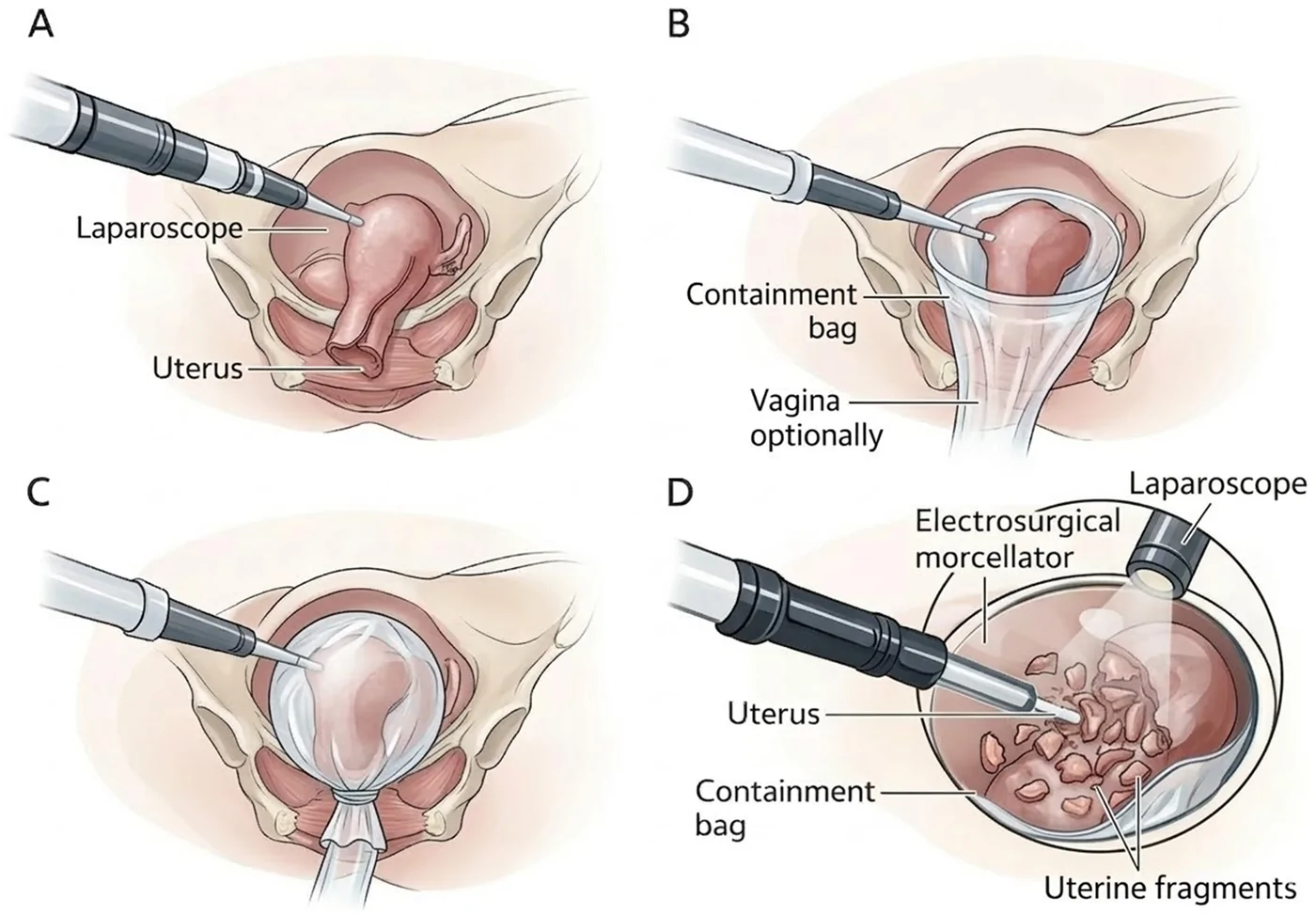
Schematic illustration of the transvaginal in-bag morcellation procedure. **(A)** The exceedingly large uterus is completely mobilized via laparoscopy. **(B)** The high-toughness containment bag is introduced transvaginally to completely encapsulate the uterus. **(C)** The uterus is securely enclosed within the bag, and pneumo-bag is established. **(D)** Under direct visualization, an electromechanical morcellator is introduced to systematically perform volume reduction on the peripheral myometrial walls, with all specimen fragments retained within the bag.

The high-strength isolation bag and supportive surgical access instruments used in this study are shown in Figure 1. A highly durable, sterile Laparoscopic Sealed Morcellation Containment Bag (Model: HB-DY; Hefei Hebo Medical Device Co., Ltd., China) was introduced transvaginally. To accommodate the massive specimens, a high-capacity bag (approximately 3000 mL) made of transparent thermoplastic polyurethane (TPU) was utilized. This TPU material provides excellent puncture resistance against the morcellator blade, while its transparency ensures optimal light transmission and visibility within the pelvic cavity. To facilitate visual and operational access, the bag is designed with integrated multi-port sleeves featuring detachable sealing caps. Finally, the electromechanical morcellator (Tissue Circular Knife, Model: HB-QD; Hefei Hebo Medical Device Co., Ltd., China)—equipped with a large 30-mm stainless-steel circular blade and operated via a foot pedal at standard cutting speeds—is introduced transvaginally via the bag's main access port. The overall surgical concept is illustrated in Figure 2.

**Figure 3:**
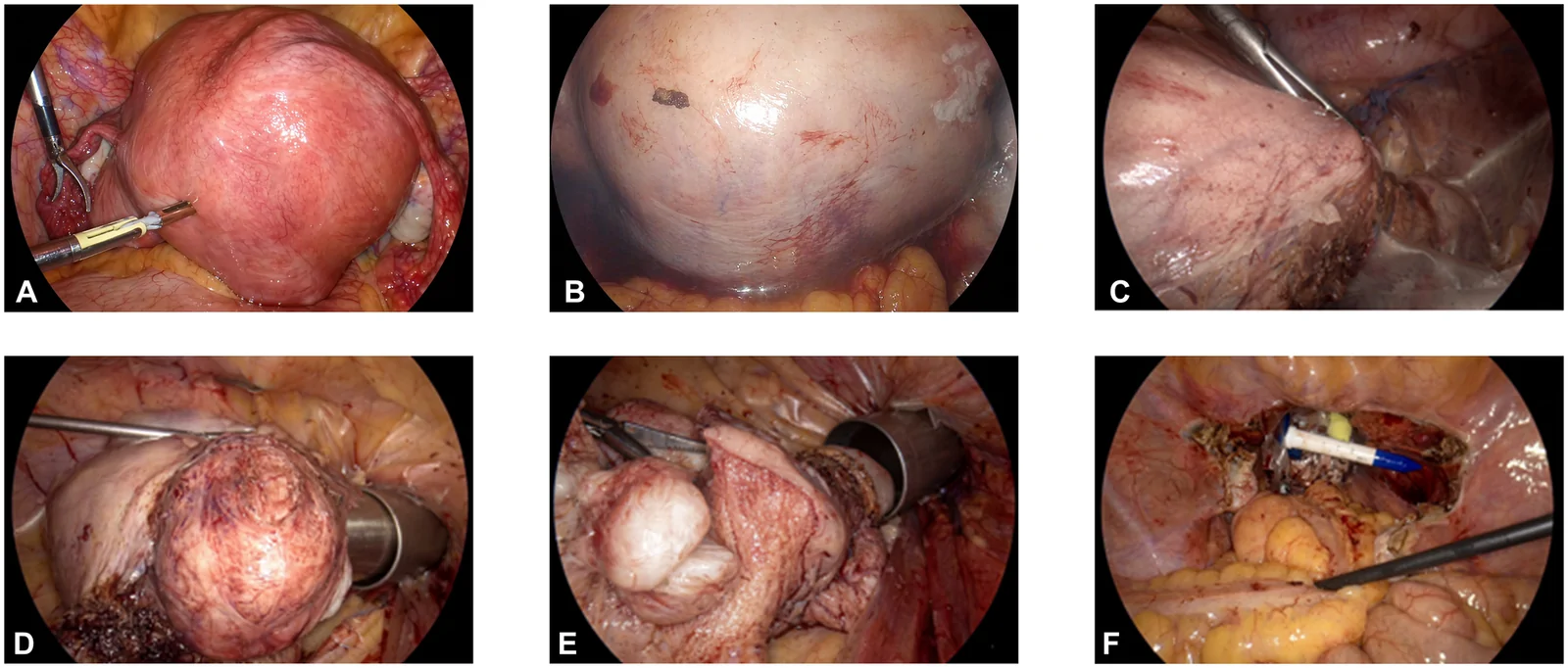
Intraoperative laparoscopic views of the surgical core steps.

**Step 1: Uterine mobilization and specimen bag insertion (Establishing the first line of defense).** The massive uterus was mobilized, and colpotomy was performed routinely under laparoscopy (Figure 3A). A high-toughness sterile polyurethane specimen bag was introduced transvaginally, and the uterus was completely placed inside the bag (Figures 2A–C). The mouth of the bag was pulled out of the vulva to establish a “transvaginal establishment of the sealed containment bag” (Figure 1C).
**Step 2: In-bag insufflation and visualization.** A trocar with an insufflation channel was inserted into the bag opening outside the body. CO2 gas was insufflated to expand the specimen bag within the pelvic cavity, providing a spacious and safe operative space inside the bag (Figure 3B). To establish visual and operational access, the bag is designed with specific sleeves featuring detachable sealing caps that can be directly docked with the abdominal trocar sheaths (Supplementary Figure A1).To clearly illustrate this complex spatial setup, Supplementary Video 1 provides a synchronized *ex vivo* and *in vivo* demonstration of the bag insertion and the establishment of the multi-port pneumo-bag system. Specifically, the sleeve marked with a yellow cap (Supplementary Figure A1b) is exteriorized and connected to the umbilical trocar (Supplementary Figure A1a) to accommodate the laparoscope for continuous intra-bag visualization. Concurrently, the sleeve marked with a blue cap (Supplementary Figure A1c) is connected to a lateral working trocar for auxiliary surgical maneuvers. The electromechanical morcellator is then introduced transvaginally via the bag's main access port.
**Step 3: Core Innovation—Four-wall directional morcellation (Preserving endometrial integrity).** To ensure procedural reproducibility, the directional morcellation followed a standardized clinical workflow based on anatomical landmarks and dual-guidance (visual and tactile). Preoperative imaging (ultrasound/MRI) was routinely reviewed to mentally map the spatial deviation of the endometrial cavity. Intravaginally, the cervix (or lower uterine segment) was firmly grasped with a tenaculum to establish a fixed cervical-fundal traction axis. Under continuous direct laparoscopic visualization from above, the electromechanical morcellator was introduced (see Supplementary Video A2 for a dynamic demonstration of the *in vivo* directional morcellation process). The cutting direction was strictly maintained parallel to this traction axis, systematically shaving off the peripheral myometrium along the anterior, posterior, left, and right walls.
Crucially, when managing severely distorted uteri with uneven thickness or multiple fibroids, the surgeon adhered to the principle of retaining a protective ‘myometrial mantle.’ The objective was targeted volume reduction rather than complete enucleation. The morcellator was used to shave off the prominent outer myometrium and subserosal fibroids. If deep intramural or submucosal myomas were encountered, the surgeon refrained from deep, aggressive morcellation to avoid cavity breach. Instead, these deep lesions were deliberately left intact within the central tissue. Consequently, the massive uterus was effectively debulked, leaving a thick, protective core housing the intact endometrial cavity.
**Step 4: En bloc extraction and macroscopic verification.** The morcellated myometrial fragments and the intact “core cavity” were pulled out of the specimen bag together. The specimen bag was then removed, and the vaginal fornix was sutured routinely (Figure 3F). Postoperatively at the back table, successful preservation was defined and confirmed macroscopically: the extracted uterine core was opened using a classic Y-shaped incision (from the cervix extending upward and branching toward both cornua) to optimally expose and visually inspect the endometrial lining. Structural continuity of the mucosa was verified to ensure no mechanical breaches or blade perforations had occurred into the cavity.

### 2.3 Statistical analysis

Statistical analysis was performed using GraphPad Prism 10.0 software. Continuous variables were expressed as means ± standard deviations (SD).

## 3 Results

### 3.1 Initial surgical outcomes

All 25 complex hysterectomies for exceedingly large uteri were successfully completed by the same senior surgical team using this technique, with no conversions to laparotomy. The baseline demographic and anatomical characteristics of the patients in this cohort are detailed in Table 1. Regarding clinical presentations, 7 patients sought surgical intervention primarily due to abnormal uterine bleeding (AUB), presenting with either multiple fibroids or adenomyosis. The remaining 18 patients presented predominantly with severe dysmenorrhea (associated with adenomyosis) or bulk/compression symptoms (associated with large fibroids). Consequently, only the 7 patients with AUB underwent strict preoperative endometrial evaluation prior to TLH. Specifically, the mean preoperative uterine size was 15.2 ± 1.8 gestational weeks. Regarding the etiology of the massively enlarged uteri, 19 patients presented with multiple uterine fibroids (mean number of nodules: 7.5 ± 3.4). Given the high multiplicity of the nodules, these patients typically exhibited a complex, mixed distribution of fibroids. Among the 19 patients with fibroids, intramural involvement (FIGO types 3-5) was present in all cases (19 cases). Furthermore, concurrent submucosal (FIGO types 0-2) and subserosal (FIGO types 6-7) fibroids were observed in 8 and 12 cases, respectively, with 5 cases harboring all three classifications simultaneously. Additionally, 11 patients (44.0%) were diagnosed with adenomyosis in the total cohort, among whom 5 patients exhibited coexisting multiple fibroids and adenomyosis.

**Table 1:** Baseline clinical characteristics of patients (*n* = 25).

Clinical Parameter	Value (Mean ± SD or n, %)
Mean Age (years)	46.5 ± 5.2
Body Mass Index (BMI, kg/m ^2^)	25.4 ± 3.6
Primary Disease Etiology	
Multiple uterine fibroids only	14 (56.0%)
Severe adenomyosis only	6 (24.0%)
Coexisting fibroids and adenomyosis	5 (20.0%)
Preoperative Uterine Size Assessment (Gestational Weeks)	15.2 ± 1.8
Subgroup: Multiple uterine fibroids only (*n* = 14)	15.5 ± 1.9
Subgroup: Severe adenomyosis only (*n* = 6)	14.1 ± 1.2
Subgroup: Coexisting fibroids and adenomyosis (*n* = 5)	15.8 ± 1.5
Preoperative Estimated Uterine Volume (cm^3^)	725.6 ± 215.4
Uterine Volume Range (cm^3^) ^a^	400–1250
Number of Fibroids (in patients with fibroids) ^b^	7.5 ± 3.4
Concurrent Fibroid Distribution (FIGO classification) ^c^	
Patients with Submucosal involvement (FIGO types 0-2)	8 (42.1%)
Patients with Intramural involvement (FIGO types 3-5)	19 (100%)
Patients with Subserosal involvement (FIGO types 6-7)	12 (63.2%)
Specific Overlap Subgroups:	
Confined to types 3-5 only	4 (21.1%)
Coexisting types 0-2 and 3-5	3 (15.8%)
Coexisting types 3-5 and 6-7	7 (36.8%)
Coexisting types 0-2, 3-5, and 6-7	5 (26.3%)
Primary Surgical Indications (Symptoms)	
Abnormal uterine bleeding (AUB)	7 (28.0%)
Dysmenorrhea and/or bulk/compression symptoms	18 (72.0%)
Preoperative Endometrial Evaluation performed	7 (28.0%)

a Calculated using the ellipsoid formula: Uterine Volume (cm^3^) ≈ 0.523 × Length (cm) × Width (cm) × Thickness (cm); where length, width, and thickness were measured from preoperative imaging (13).

b Evaluated in the 19 patients presenting with multiple uterine fibroids (14 with fibroids only, 5 with coexisting adenomyosis).

c Evaluated in the 19 patients with fibroids. Because patients presented with multiple nodules (mean 7.5), a single patient frequently harbored fibroids of multiple FIGO classifications simultaneously. Percentages in this section are calculated based on the 19 patients with fibroids.

### 3.2 Preliminary assessment of extraction efficiency: observations on uterine size and morcellation time

Notably, although the mean uterine volume exceeded 700 cm^3^, the mean time for transvaginal in-bag four-wall morcellation was 12.5 ± 4.2 min. Clinical observation revealed that the larger the uterus (especially in cases of adenomyosis or multiple fibroids with extremely thickened peripheral myometrium), the longer the safe distance for the electromechanical morcellator to perform volume reduction on the four walls. Consequently, the directional morcellation could be smoothly performed on the four walls, demonstrating the technical feasibility of extracting massive uteri within the containment bag.

Postoperative systemic evaluation of surgery-related indicators and specimen integrity was conducted (Table 2).

**Table 2:** Surgical outcomes and anatomical integrity evaluation indicators (*n* = 25).

Surgical and Recovery Parameter	Value or Percentage
Conversion to Open Surgery Rate	0% (0/25)
Average In-Bag Morcellation Time (min)	12.5 ± 4.2
Intraoperative Estimated Blood Loss (mL)	40.5 ± 12.0
Rate of Preserved Endometrial Physical Structure	100% (25/25)
Postoperative Specimen Bag Methylene Blue Pressure Test Without Leakage	100% (25/25)
Rate of Abdominal/Pelvic Organ or Vaginal Wall Injury	0% (0/25)
Postoperative Average Hospital Stay (days)	2.1

### 3.3 Endometrial integrity assessment

**Figure 4:**
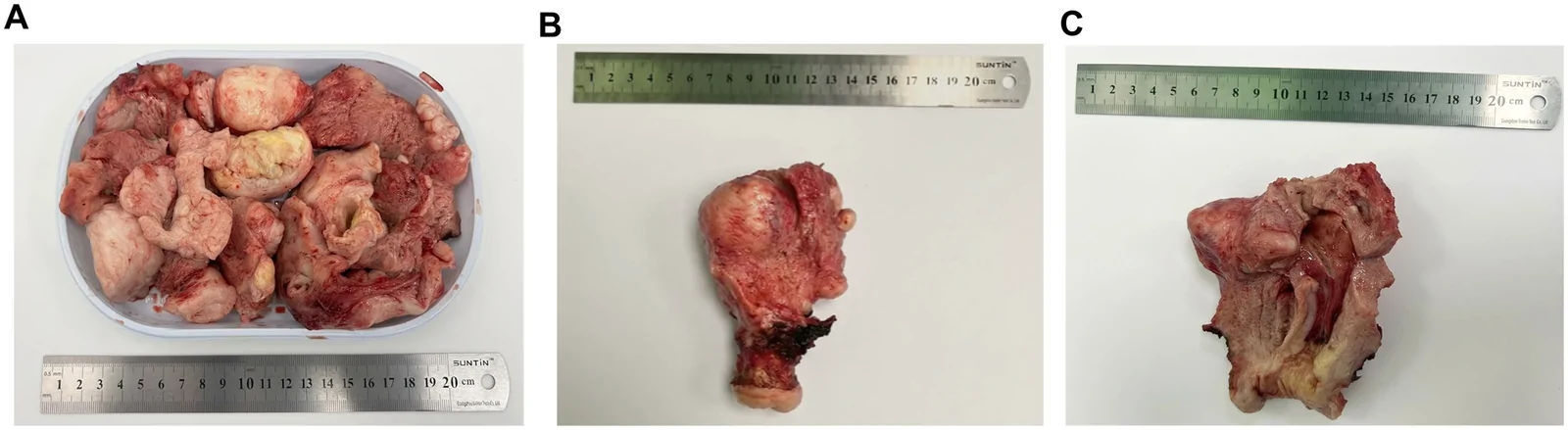
Postoperative macroscopic evaluation of the extracted uterine specimen. **(A)**The complete collection of extracted tissues, including a large volume of morcellated peripheral myometrial fragments and the central core with its integrity maintained. **(B)** Close-up view of the central “core cavity” specimen, demonstrating no disruption to its external structure. **(C)** Cross-sectional view of the incised core specimen, confirming that the anatomical structure of the endometrial layer achieved 100% integrity, with no mechanical disruption or perforation.

Postoperative dissection of all 25 extracted specimens included a large number of peripheral myometrial fragments and a well-preserved central “core cavity” (Figures 4A,B). Incision of the core specimen confirmed that the endometrial layer was macroscopically structurally intact (Figure 4C), with no cases of premature mechanical disruption or perforation during the morcellation process.

### 3.4 Sealed bag test

**Figure 5:**
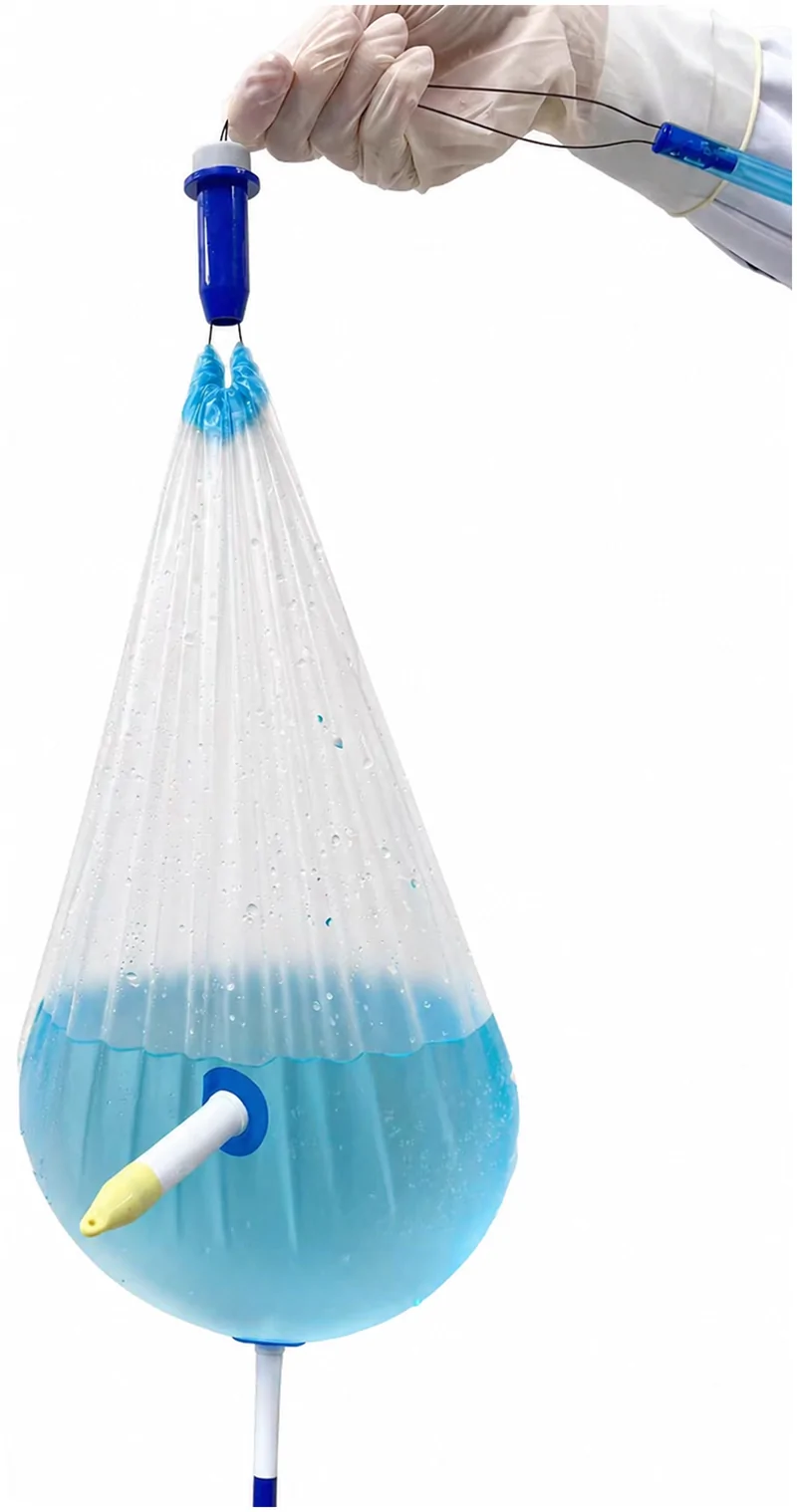
Postoperative pressure test of the specimen bag. Following specimen extraction, the bag is suspended and injected with methylene blue-dyed normal saline for a pressure test. The results confirm that the specimen bag demonstrated fluid-tight integrity without macroscopic punctures, with no punctures or leakage occurring, thereby ensuring the complete isolation of surgical contents from the abdominopelvic cavity.

Crucially, there were zero instances of intraoperative technical device failures, and the electromechanical morcellator functioned smoothly in all cases. At the end of the surgery, the extracted specimen bags were subjected to a suspended pressure test using normal saline containing methylene blue (Figure 5). The test confirmed that 25 (100%) of the specimen bags maintained fluid-tight integrity without any punctures or leaks, achieving complete isolation of uterine cavity contents within the abdominopelvic cavity.

### 3.5 Postoperative recovery

The mean estimated blood loss was 40.5 ± 12.0 mL. No adjacent organ injury (bowel, urinary system) or vaginal wall thermal injury occurred in the entire cohort. The mean length of hospital stay was 2.1 days, reflecting favorable Enhanced Recovery After Surgery (ERAS) outcomes.

## 4 Discussion

For the minimally invasive resection and specimen extraction of exceedingly large uteri, mitigating the risk of tissue dissemination while maintaining technical feasibility has always been a dual challenge in clinical gynecology. Historically, the minimally invasive extraction of exceedingly large uteri has evolved through several stages. In the early era of laparoscopic hysterectomy, excessively large specimens often required enlargement of the abdominal incision or conversion to laparotomy because intact transvaginal extraction was technically difficult. Although effective for specimen removal, these approaches partially compromised the advantages of minimally invasive surgery.

Subsequently, various transvaginal fragmentation and morcellation techniques were introduced to avoid laparotomy. During traditional transvaginal manual extraction, the massive volume of the specimen is often the primary factor leading to a cramped pelvic space, prolonged surgical time, and increased complications (12–14). More importantly, increasing evidence demonstrated that uncontained morcellation could disseminate occult malignant cells or endometrial tissue, potentially resulting in parasitic leiomyoma formation, disseminated peritoneal leiomyomatosis, iatrogenic endometriosis, or unexpected oncological upstaging (4, 7, 10). These concerns ultimately led to the FDA warning regarding uncontained power morcellation.

Following this paradigm shift, “contained” or “in-bag” morcellation gradually became an international trend in minimally invasive gynecology. Previous studies demonstrated the feasibility of manual or power morcellation within a containment bag (3, 15, 16). However, currently reported techniques still have several limitations, including prolonged operative time during manual morcellation, limited visualization during electromechanical morcellation, and insufficient attention to potential biological dissemination caused by disruption of the endometrial cavity.

To better understand the clinical positioning of our proposed method amidst these existing options, a brief comparison with currently published extraction techniques is warranted:

(1) Traditional Transvaginal Manual Morcellation: As comprehensively reviewed by Donat, uncontained manual vaginal morcellation using a cold scalpel has been a historically feasible alternative to laparotomy, successfully applied to uteri weighing over 1000 g (17). Its primary advantage lies in its simplicity, requiring no specialized containment bags or power equipment. However, this traditional approach has notable drawbacks: it is highly time-consuming, ergonomically exhausting for the surgeon, and relies heavily on forceful traction and blind or semi-blind sharp dissection within the narrow vaginal canal, which inherently carries the risk of adjacent organ injury. Furthermore, uncontained manual morcellation still carries a documented risk of inadvertently disseminating occult malignant cells or benign lesional tissue into the pelvic cavity or vaginal walls.
(2) Standard Power In-bag Morcellation (Transabdominal/Transumbilical): As reported by Cohen power morcellation within an insufflated bag significantly reduces operative time and physical fatigue (15). Yet, the primary drawback is the random fragmentation of the specimen. This complete destruction of anatomical orientation severely complicates the pathologist's ability to map the depth of invasion if an occult malignancy is discovered. Additionally, the blind plunging motion of the rapidly spinning blade carries a high risk of inadvertently piercing the containment bag.
(3) Transvaginal Four-wall Directional In-bag Morcellation (Our Technique): Compared to these methods, our technique seeks a functional middle ground. By utilizing an electromechanical device, we maintained a highly efficient mean in-bag morcellation time of 12.5 minutes for uteri averaging 725 g, effectively overcoming the physical exhaustion and prolonged operative times associated with traditional manual vaginal extraction (16, 17). By conducting the procedure entirely within a sealed pneumo-bag under direct laparoscopic visualization, we mitigate the risks of both tissue dissemination and blind vaginal injury. This illuminated, magnified visual field transforms a traditionally blind, forceful extraction into a highly controlled, precise directional debulking process. Most importantly, by adopting a directional ‘outside-in’ shaving approach rather than random fragmentation, we overcome the pathological disadvantages of standard power morcellation, preserving an intact central ‘core’ for accurate diagnosis. However, the inherent disadvantages of our technique include a steep learning curve, strict dependence on specialized multi-port containment bags, and the requirement for advanced hand-eye coordination under diminished tactile feedback.

The safe execution of this directional strategy capitalizes on the intrinsic anatomical features of the exceedingly large uterus. The hyperplastic portion of a massive uterus usually originates from the thickened peripheral myometrium or multiple leiomyomas, and this technique specifically creates morcellation channels around the uterine periphery. Theoretically, the thicker the peripheral myometrium, the deeper and wider the physical buffer zone for the safe operation of the morcellator, thereby mitigating the risk of accidentally injuring the central endometrial cavity or puncturing the specimen bag. This outside-in, layer-by-layer volume reduction approach allows the massive specimen to be orderly reduced in a controlled manner, which demonstrated procedural feasibility and provided a systematic operational workflow for the surgeon.

Regarding safety, this exploratory study tested a “dual barrier” theoretical model at the technical level aimed at preventing iatrogenic dissemination. The first line of defense is the “anatomic barrier”—namely, the preservation of the relative integrity of the uterine cavity. Traditional disordered morcellation carries a high risk of leading to cavity rupture, causing tissue fluid containing endometrial cells to spill; in contrast, through precise peripheral morcellation, this technique substantially preserves the “central anatomic structure” wrapping the intact endometrial cavity, thereby mitigating the risk of potential lesional cells spilling along with endogenous fluid from the source. The second line of defense is the “physical barrier”—namely, the application of an insufflated, sealed specimen bag. The high-strength polyurethane bag establishes a physical isolation layer between the operative area and the abdominopelvic cavity. The CO2 pneumo-environment established within the bag not only effectively expands the bag walls to provide a visual operative space and avoid instrument injury but also confines the blood, tissue fragments, and potential aerosols generated during the procedure strictly within the bag.

The potential for iatrogenic tumor dissemination during minimally invasive uterine surgery is a critical concern that extends beyond morcellation to any form of mechanical uterine manipulation. For instance, a recent propensity score-matched study by Yalcin et al. evaluated the oncological safety of using intrauterine manipulators during laparoscopic hysterectomy for endometrial cancer (18). Although their clinical context focused on known malignancies and a different mechanical instrument, both scenarios underscore the critical question of how mechanical forces applied to the uterus might influence the peritoneal spread of malignant cells. Given that the physical stress exerted by an electromechanical morcellator is substantially more intense than that of a standard manipulator, adopting a stringent physical containment strategy (such as our dual-barrier approach) becomes even more imperative to counteract the inherent risks of mechanical tumor dissemination during specimen extraction.

The necessity of this strict containment is fundamentally rooted in the inherent limitations of preoperative diagnostics. In standard clinical practice, routine invasive endometrial sampling is primarily indicated for patients with abnormal uterine bleeding (AUB), leaving a ‘diagnostic blind spot’ for asymptomatic patients presenting solely with bulk symptoms. Furthermore, even comprehensive preoperative screening (including biopsies and MRI) lacks sufficient sensitivity to definitively rule out occult leiomyosarcomas originating deep within the myometrium, or early-stage focal endometrial cancers hidden within a massively distorted cavity. Because the risk of unexpected occult malignancies in asymptomatic patients can never be reduced to absolute zero, unprotected morcellation carries a catastrophic risk of disease upstaging. Therefore, our ‘dual-barrier’ technique operates on the principle of ‘universal surgical precautions.’ By proactively assuming every large uterus may harbor undiagnosed pathology, this technique utilizes surgical physical containment to compensate for the inevitable uncertainties of preoperative diagnostics.

Importantly, however, our cohort consisted exclusively of benign cases, and no peritoneal washings or cytological evaluations were performed to assess for microscopic tissue dissemination. Therefore, the oncological benefits of this ‘dual-barrier’ concept must be interpreted strictly as a theoretical design rationale rather than a proven clinical benefit at this stage. Nevertheless, from a purely anatomical and surgical perspective, achieving macroscopic physical containment (an intact endometrial core within a sealed, water-tight bag) serves as a proactive fail-safe designed to minimize tissue spillage during mechanical volume reduction.

Furthermore, by avoiding abdominal incision extension, this technique preserves the aesthetic advantages of NOSE (19). The direct-vision, controllable, and sealed environment provides a structured extraction process, presenting a feasible alternative to the conventional transvaginal traction of massive specimens. This process also coincides with the core concepts of Enhanced Recovery After Surgery (ERAS), which aim to reduce surgical trauma stress and promote postoperative recovery.

In summary, transvaginal four-wall directional morcellation within a sealed bag with “preserved endometrial integrity” provides a promising preliminary exploration for managing massive uterine specimens. This strategy relies on the local anatomical features of the exceedingly large uterus to facilitate volume reduction while aiming to mitigate potential iatrogenic tissue dissemination through the construction of dual anatomic and physical barriers. With the deepening of future large-sample prospective studies, this technique is expected to provide robust clinical evidence and reference strategies for the minimally invasive and standardized extraction of massive uteri in gynecology.

## 5 Limitations

The preliminary clinical results demonstrate promising application potential; however, consistent with the nature of an initial feasibility study, certain limitations must be acknowledged.

First, the observational, single-arm design of this retrospective cohort precludes a direct comparative analysis. Without a parallel control group utilizing traditional transvaginal or transabdominal extraction methods, any comparative superiority regarding extraction efficiency or complication rates cannot be definitively established at this stage. Second, the sample size (*n* = 25) is relatively small. While it is sufficient to evaluate the primary mechanical endpoints—such as successful in-bag morcellation and macroscopic bag integrity—it is underpowered to detect rare intraoperative adverse events, meaning the full safety profile requires validation in larger cohorts. Third, our assessment of specimen containment and endometrial integrity relies exclusively on macroscopic parameters (visual inspection and methylene blue leakage test). We acknowledge that macroscopic intactness does not equate to microscopic or cellular containment. Our study lacked patients with occult malignancies, omitted intraoperative peritoneal cytology to detect microscopic spillage, and lacked long-term survival follow-up. Most critically, because asymptomatic patients in this cohort did not undergo routine preoperative endometrial evaluation—reflecting real-world clinical constraints—we cannot definitively ascertain whether subclinical or premalignant endometrial changes were present. Consequently, the actual capability of the preserved ‘endometrial core’ to withstand the spillage of biologically active, malignant cells under the physical stress of morcellation remains untested. Therefore, any claims regarding ‘oncological safety’ or the efficacy of the ‘dual-barrier’ strategy against tumor dissemination are purely theoretical concepts and hypothesized advantages, not proven clinical efficacies. Fourth, from a purely surgical perspective, this technique entails a specific learning curve. Operating an electromechanical morcellator within the confined pneumo-environment of an isolation bag alters spatial orientation and requires surgeons to adapt to diminished tactile feedback. Because the 25 cases in this cohort were performed concurrently by multiple attending physicians at our center, a single linear learning curve regarding operative time across consecutive cases could not be statistically evaluated. Nevertheless, the successful independent completion of these procedures by multiple attendings suggests that the learning curve is manageable. Regarding clinical applicability, the reproducibility of this technique does not rely on the unique expertise of a single operator, but it does require a baseline proficiency in advanced endoscopic skills. It is not intended as a universal replacement for standard extraction of moderately enlarged uteri, but rather serves as a valuable, visually-controlled alternative for safely managing exceedingly large and difficult uterine specimens for surgeons well-versed in conventional laparoscopic hysterectomy.

To overcome these methodological limitations and transition from technical feasibility to high-level clinical evidence, our research team is actively conducting a prospective, multicenter, large-sample randomized controlled trial (Clinical Trial Registration Number: NCT07486622). This ongoing trial aims to systematically evaluate the long-term oncological safety, comprehensive clinical efficacy, and generalizability of this technique.

## 6 Conclusion

In conclusion, this study represents a promising preliminary clinical exploration of “transvaginal four-wall directional in-bag morcellation” for the extraction of exceedingly large uteri during minimally invasive surgery. By establishing an innovative “dual barrier” defense system—comprising the anatomical preservation of the endometrial cavity and the physical isolation of a sealed specimen bag—this study strictly explores the operational viability of this approach. Our initial results demonstrate that the procedure is technically feasible, successfully providing intact macroscopic physical containment during the extraction of massive uteri without comparative claims to efficiency. However, as a retrospective proof-of-concept cohort study with a limited sample size, these findings are strictly preliminary. Further validation through ongoing large-scale, multicenter, prospective randomized controlled trials is imperative to systematically evaluate its reproducibility, comparative complication rates, microscopic bag integrity, and long-term oncological safety in gynecology.

## Data Availability

The raw data supporting the conclusions of this article will be made available by the authors, without undue reservation.
