# Supplementary material for: Transvaginal in-bag directional morcellation with endometrial preservation for large uteri: an initial clinical feasibility study
Source: Front Med (Lausanne). 2026 Sep 9;13:1910837. doi: 10.3389/fmed.2026.1910837 (PMC13597801; doi:10.3389/fmed.2026.1910837)
Supplement: Supplementary file 1 [file Table1.docx]

Supplementary Material

# 1 Supplementary Data

**Figure A1.** Detailed schematic and intraoperative views of the in-bag trocar docking system.

Following transvaginal placement of the isolation bag, its operational sleeves are connected to the abdominal ports. The detachable sealing caps allow direct docking with standard trocar sheaths. The sleeve with the yellow cap (b. Laparoscope trocar) is pulled through the abdominal wall and docked with the umbilical trocar (a. Umbilical Trocar) to establish the visual channel. The sleeve with the blue cap (c. Working trocar) is docked with an auxiliary port for instrument access. The electromechanical morcellator is introduced through the main transvaginal port of the bag.

**Supplementary Video A1.** A synchronized ex vivo (in vitro) and in vivo demonstration illustrating the transvaginal insertion of the containment bag, spatial orientation, and the establishment of the multi-port pneumo-bag system.

**Supplementary Video A2.** An in vivo surgical demonstration of the transvaginal four-wall directional morcellation technique following uterine detachment, highlighting the systematic "outside-in" shaving process while preserving the central endometrial core.
